# A New Cause of Tabes

**Published:** 1891-02-07

**Authors:** 


### A NEW CAUSE OF TABES.

Bernhardt reports in the *Neurol. Centralbl.*, Dec., 1890, a case of locomotor ataxy in a young woman, in which cutaneous sensibility was intact, but the loss of muscular sense and of control over the movements of the limbs almost complete. No cause whatever could be found to account for the disease except that she had worked a heavy pedal sewing-machine from early morning till near midnight. Bernhardt quotes two similar cases from Guelliot. Under the circumstances of the age of the patients, the positive exclusion of syphilis, and the special character of the ataxy, the explanation is highly probable, and deserves further consideration now that so many thousands of women are thus employed.

